# Radiographic and Ultrasonographic Study of the Etiology of High‐Rise Syndrome in Cats: A Retrospective Analysis

**DOI:** 10.1002/vms3.71002

**Published:** 2026-05-19

**Authors:** Parisa Haghi, Sarang Soroori, Mohammad Molazem, Mir Sepehr Pedram, Athena Salimi, Arezoo Ramezani

**Affiliations:** ^1^ Faculty of Veterinary Medicine University of Tehran Tehran Iran; ^2^ Surgery and Radiology Department, Faculty of Veterinary Medicine University of Tehran Tehran Iran

**Keywords:** cats, etiology, high‐rise, radiography, retrospective, sonography, syndrome

## Abstract

**Background:**

High‐rise syndrome (HRS) is a common traumatic presentation in cats.

**Objective:**

This study evaluated the etiology, frequency and types of radiographic and sonographic lesions associated with HRS in 120 cats.

**Methods:**

Information regarding the circumstances of the falls was collected from the owners. The data were analysed to identify causative factors, types and frequency of injuries.

**Results:**

It was found that most affected patients were under 1 year old and the majority of cases fell from the third floor. Approximately 75% of cats had a history of high‐risk behaviours, as reported by the owners. Femoral fractures were the most frequent skeletal injury (17.5%), while thoracolumbar spinal injuries (T13‐L1) were observed in 50% of cases of vertebral column injuries. Pulmonary contusions and haemorrhage were the most common soft tissue injuries, occurring in 45% of cases. No significant correlation was found between fall height and injury severity; however, higher injury severity was associated with increased mortality. The overall survival rate was 92.5%.

**Conclusion:**

Preventive measures, such as restricting access to windows and balconies, are recommended to reduce the incidence of HRS.

## Graphical abstract

This retrospective study analysed the etiology and imaging (radiographic/ultrasonographic) findings in 120 cats with high‐rise syndrome. Most affected cats were under 1 year old and fell from the third floor. The survival rate was 92.5%. Pulmonary contusions were the most frequent soft tissue injury, occurring in 45% of cases

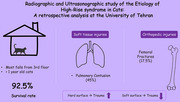

## 1 Introduction

High‐rise syndrome (HRS) refers to injuries sustained from a fall of two or more floors (approximately 7 m or 23 ft) and may result in significant trauma or death (Boudrieau 2004; Buriko 2018; Cruz and Nykamp 2012; Kapatkin and Matthiesen 1991; Kazarian et al. 1976; Reiter 2007). HRS in cats is a common presentation in veterinary emergency practice (Battaglia and Steele 2016; Collard et al. 2005; Merbl et al. 2013; Uzun et al. 2023). Cats with HRS usually fall from balconies, windows or rooftops (Flagstad et al. 1999). Falls are often associated with playful behaviour, curiosity, hunger, loneliness or environmental factors such as outdoor birds, lack of suitable toys or slippery surfaces (Oxley and Montrose 2016; Warner and Demling 1986). Young cats, particularly those between 1 and 3 years old, are more frequently affected (Gordon et al. 1993; Warner and Demling 1986). Historically, HRS was characterized by a triad of epistaxis, cleft palate and pneumothorax; later studies expanded this definition to include limb fractures (Gawor and Niemiec 2021; Garosi and Adamantos 2011; Liehmann et al. 2012; Papazoglou et al. 2001; Rahman et al. 2015; Reiter 2007; Vidal et al. 2024; Vnuk et al. 2004; Whitney and Mehlhaff 1987). Previous studies have analysed the relationships between age, sex, sterility status, time of day, height, breed, month and season and severity and frequency of injuries (Bonner et al. 2012; Girol‐Piner et al. 2022; Karabağlı et al. 2024; Oxley and Montrose 2016; Tüzün and Sağlam 2009; Vidal et al. 2024; Xiang 2018).

The present study aims to retrospectively analyse the etiology (number of storeys that cats fell, sex, reproductive status, landing surface, etc.) of HRS in cats and characterize the frequency and types of injuries observed in radiographic and sonographic examinations of 120 cases over a 12‐month period at the Small Animal Hospital.

## 2 Materials and Methods

This retrospective study included 120 cats presented to the Radiology Department between 21 March 2023 and 21 March 2024, with a history of HRS. Stray cats were excluded due to incomplete medical records. Clinical information was obtained from the Hospital Information System (HIS) and the Picture Archiving and Communication System (PACS). Additional data, including age, sex, reproductive status, date and time of fall, fall circumstances, previous falls, risky behaviours, household conditions and duration of ownership, were collected from the owners. Ethical approval for this study was obtained from all cat owners and the relevant authorities. All medical data were used in an anonymized form, ensuring that no identifiable information was included in the manuscript. This retrospective study was conducted using clinical data from the Faculty of Veterinary Medicine. The data were collected under an approved DVM thesis project (thesis code: 4121; administrative notification/project number: 2379). HRS is generally defined as a fall from two or more floors. Despite the original definition by Reiter (2007), some studies, such as that by Robinson (1976), included the first floor as one of the involved floors; therefore, cats who fell from the first floor or 3 m (12 cats) were also included in this study.

Table 1 summarizes the scoring system table used to classify each patient's injuries. Each patient's injuries were recorded and scored to assess potential correlations between fall height and injury severity. The injury scoring system was adapted from Vnuk 2004. To allow a more precise assessment, injuries were classified into three categories: mild (Score 1), moderate (Score 2), and severe (Score 3), corresponding to minor, urgent and life‐threatening injuries, respectively. A score of zero was assigned to cats that sustained no injuries following a fall.

**TABLE 1:** Injury scoring system of HRS patients.

Score 0—Normal	Score 1—Mild	Score 2—Moderate	Score 3—Severe
No significant injury	Mesenteric reactive lymphadenomegaly	Pneumothorax	Skull fracture
		Pneumomediastinum	
	Subcutaneous emphysema	Pneumoabdomen	
	Mild pulmonary contusion/haemorrhage	Moderate pulmonary contusion/haemorrhage	
		Abdominal hernia	
	Epistaxis	Pleural effusion	Vertebral fractures
		Pulmonary atelectasis	
	Steatite	Abdominal free fluid	
		Splenic haematoma	
	Bulla in the lungs	Peritonitis	
		Pancreatitis	
	Microspleen	Haematuria/injury of the bladder mucosa	Vertebral displacement with neurological symptoms
		Limb fractures	
	Tooth fracture	Rib fracture	
		Joint luxation	
	Mild skin and muscle ruptures	Fracture of the mandible	
		Rupture of the collateral ligament of the knee	

## 3 Statistical Analysis

Data were statistically analysed using IBM SPSS Statistics 26 software. The analysis included one sample of Kolmogorov–Smirnov, Kruskal–Wallis and Mann–Whitney *U* tests. Statistical significance was set at *p* < 0.05.

## 4 Results

A total of 120 cases were reviewed over a one‐year period for possible injuries after falling from a height. In total, 7.5% (nine cats) of cases (with an average injury score of 15) died, and 3.3% (four cats) of cases (with an average injury score of 10.75) were euthanized due to the severity of the injuries. The overall survival rate, including euthanized cases, was 89.2% (107 patients). Excluding euthanized cases, it was 92.5% (111 cases). Notably, 5.8% (seven cats) of the cases did not sustain any particular damage.

### 4.1 Height of Fall

**FIGURE 1:**
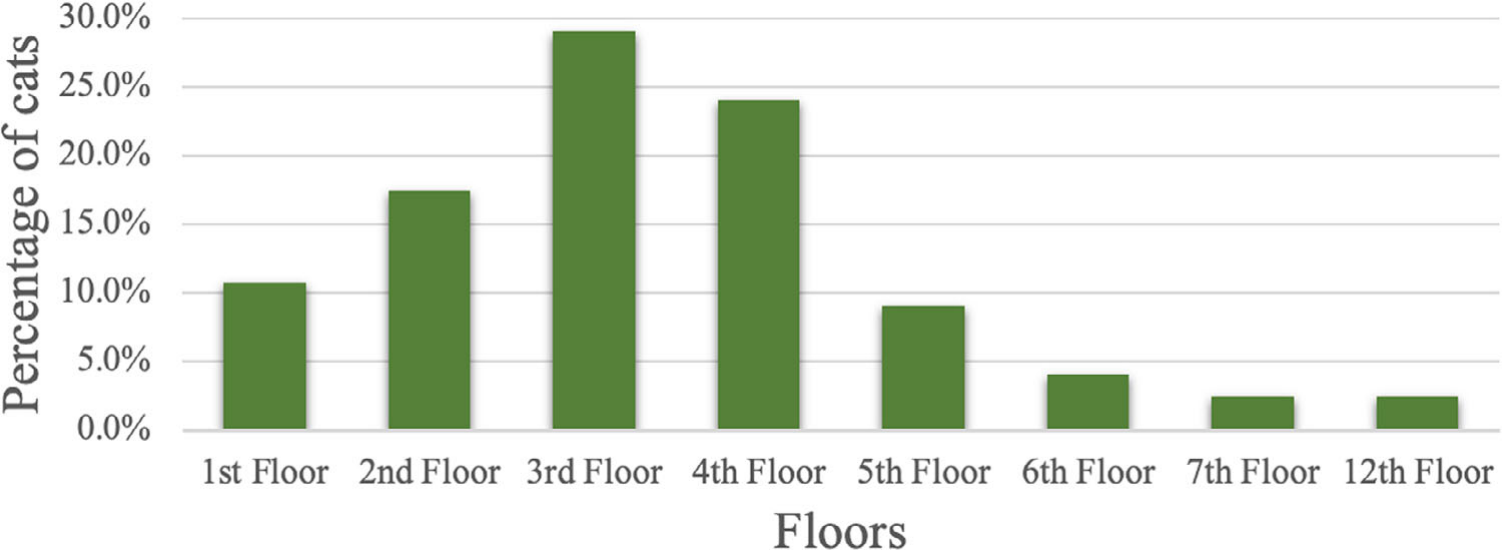
The association between storeys and number of cats that suffered from HRS.

In this study, 10.8% (13 cats) of cases fell from the 1st floor or 3 m, 17.5% (21 cats) from the 2nd floor, 30.1% (36 cats) from the 3rd floor, 24.1% (29 cats) from the 4th floor, 9.1% (11 cats) from the 5th floor, 4.1% (5 cats) from the 6th floor, 2.5% (3 cats) from the 7th floor and 2.5% (3 cats) from the 12th floor. The most frequently fallen floors were the third, fourth and second floors, respectively (Figure 1). No cat in this study fell from the 8th, 9th, 10th or 11th floors. Among cats falling from the first floor, 61.5% (eight cats) were under 1 year old.

The Kruskal–Wallis test was used to evaluate the possible relationship between fall height and injury scores as defined in this study. The *p*‐value was 0.061, which is not statistically significant, indicating no significant correlation between fall height and severity of injuries (Figure S1).

### 4.2 Age Distribution

**FIGURE 2:**
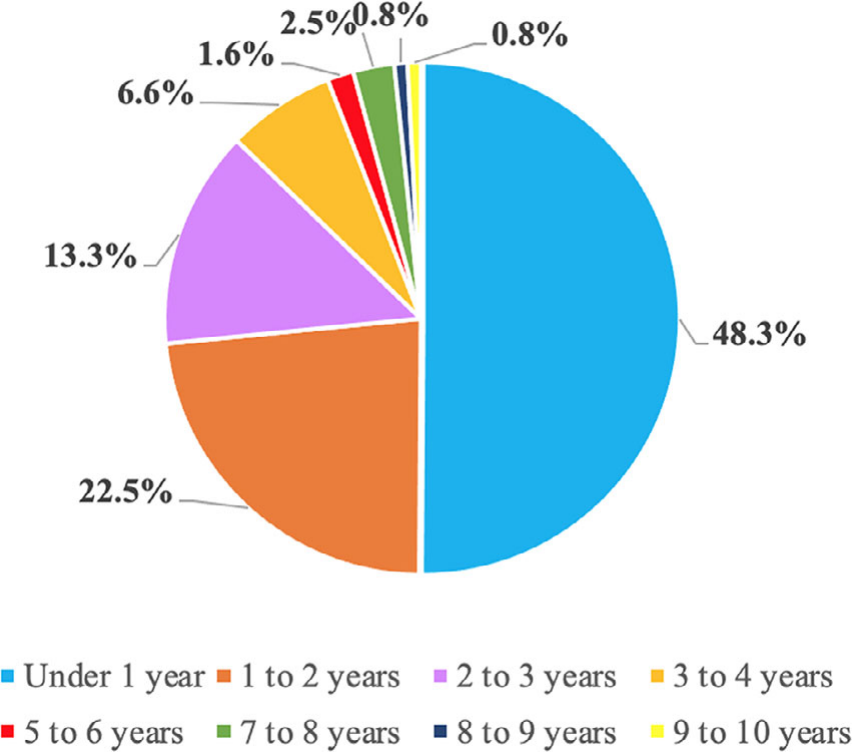
Age distribution of cats with HRS.

With a frequency of 49.3% (59 cats), the most common age of cats falling from heights was under 1 year old. Ages between 1 and 2 years were 23.5% (28 cats); between 2 and 3 years, 14.3% (17 cats); between 3 and 4 years, 6.6% (8 cats); between 5 and 6 years, 1.6% (2 cats); between 7 and 8 years, 2.5% (3 cats); between 8 and 9 years, 1% (1 cat); and between 9 and 10 years, 1% (1 cat) (Figure 2).

### 4.3 Sex and Neuter Status

**FIGURE 3:**
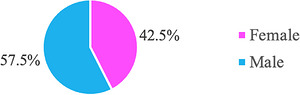
Gender distribution of cats with HRS.

**FIGURE 4:**
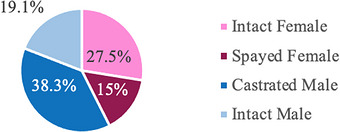
Sex and neuter status distribution of cats with HRS.

Among the 120 patients, 69 (57.5%) were male and 51 (42.5%) were female. Additionally, 38.3% (46 cats) of the cases were neutered males, 19.2% (23 cats) intact males, 15% (18 cats) spayed females and 27.5% (33 cats) intact females (Figures 3 and 4).

The Mann–Whitney *U* test was used to evaluate the relationship between the cat's sex and injury scores. The *p*‐value was 0.065. There is no statistical significance to this finding, although male cats were more frequently prone to HRS (Figure S3). The test was also applied to assess differences between neutered and intact cats, resulting in a *p*‐value of 0.295, which is not statistically significant, although intact cats were more frequently affected (Figure S4).

### 4.4 Breed

**FIGURE 5:**
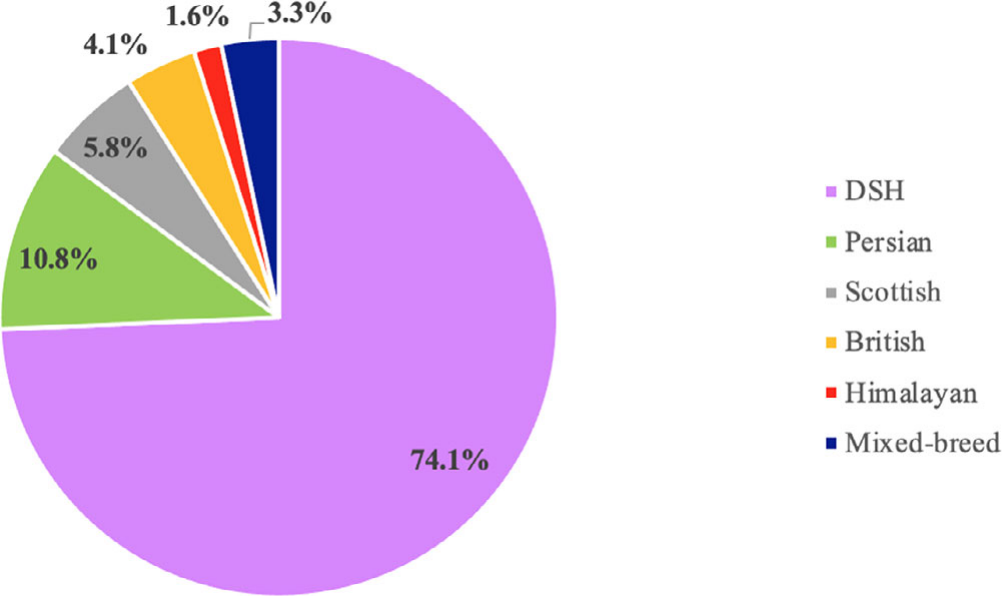
Breed distribution of cats with HRS.

As shown in Figure 5, the most common breed was Domestic Shorthair (89 cats, 74.5%), followed by Persian (13 cats, 10.8%), Scottish Fold (7 cats, 5.8%), British Shorthair (5 cats, 4.1%), Himalayan (2 cats, 1.5%) and mixed breeds (4 cats, 3.3%).

### 4.5 Seasonal and Monthly Distribution

**FIGURE 6:**
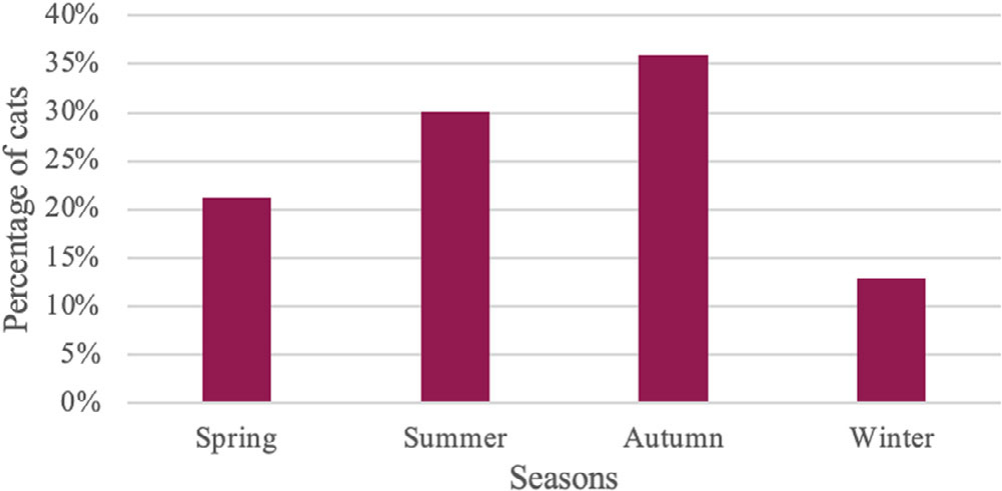
Seasonal frequency of cats with HRS.

Seasonally, 29 cats (24%) fell in spring, 36 cats (29.8%) in summer, 37 cats (30.7%) in autumn, and 18 cats (15.5%) in winter, as illustrated in Figure 6.

**FIGURE 7:**
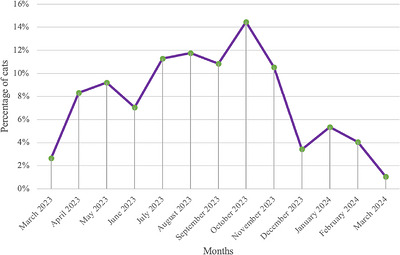
Monthly occurrence patterns of cats with HRS.

Between 21 March 2023 and 20 March 2024, the monthly distribution of HRS cases in cats was as follows: March 2023 (3 cats, 2.66%), April (10 cats, 8.32%), May (11 cats, 9.19%), June (8 cats, 7.06%), July (14 cats, 11.53%), August (14 cats, 11.53%), September (13 cats, 10.69%), October (17 cats, 14.47%), November (13 cats, 10.69%), December (4 cats, 3.42%), January 2024 (6 cats, 5.35%), February (5 cats, 4.04%) and March 2024 (1 cat, 1.05%) (Figure 7).

### 4.6 Body Weight

**FIGURE 8:**
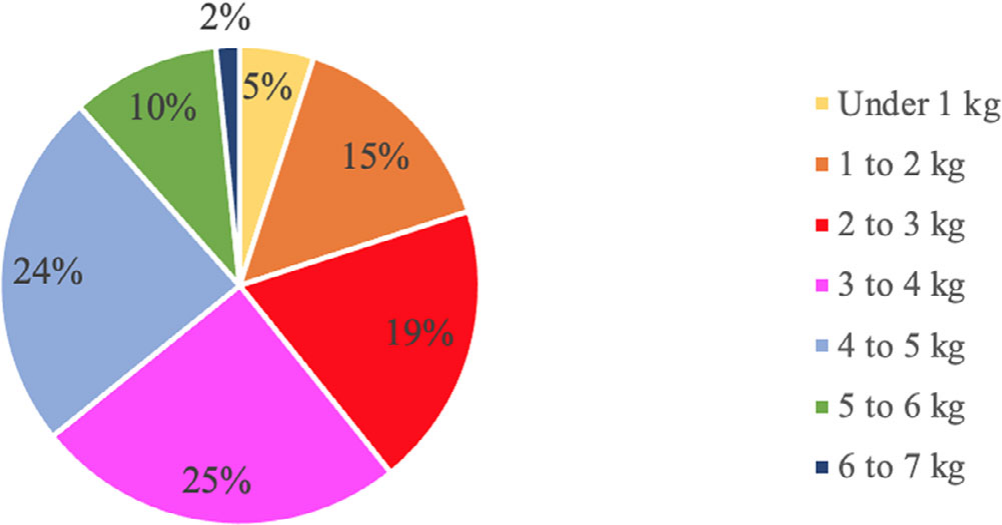
Weight frequency distribution of cats with HRS.

Regarding body weight, 6 cats (5%) weighed less than 1 kg, 18 cats (15%) between 1 and 2 kg, 23 cats (19.1%) between 2 and 3 kg, 30 cats (25%) between 3 and 4 kg, 29 cats (24.1%) between 4 and 5 kg, 12 cats (10%) between 5 and 6 kg and 2 cats (1.8%) between 6 and 7 kg, as presented in Figure 8. The Kruskal–Wallis test evaluating weight and injury scores yielded a *p*‐value of 0.089, indicating no statistically significant correlation (Figure S2).

### 4.7 Place of Fall

**FIGURE 9:**
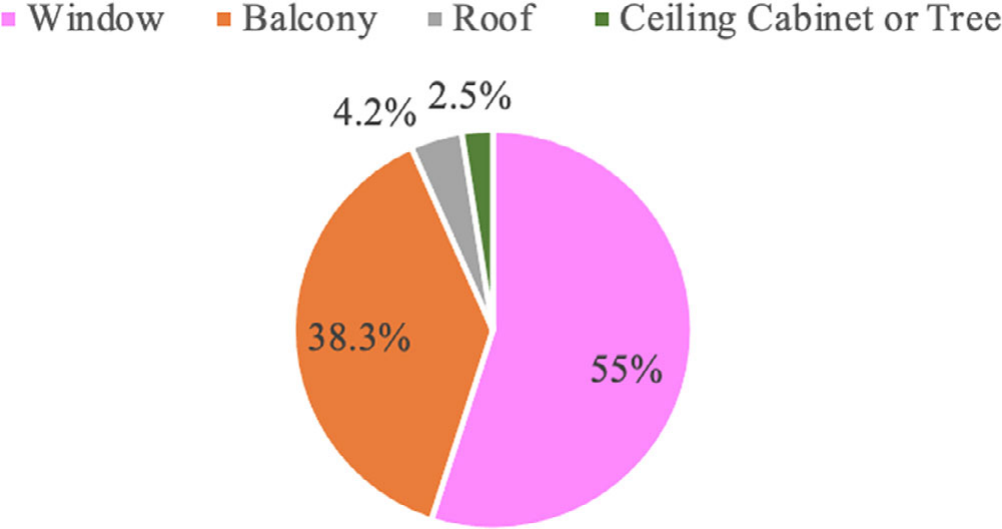
Place of incident in cats with HRS.

Overall, 66 cats (55%) fell from windows, 46 cats (38.3%) from balconies, 5 cats (4.2%) from roofs and 3 cats (2.5%) from the top of cabinets or trees (Figure 9).

### 4.8 Time of HRS

**FIGURE 10:**
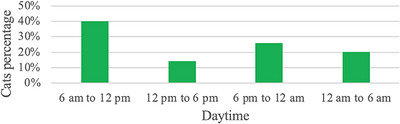
Daytime distribution of cats with HRS.

As shown in Figure 10, 48 cats (40%) fell between 6:00 AM and 12:00 PM, 17 cats (14.1%) between 12:00 PM and 6:00 PM, 31 cats (25.8%) between 6:00 PM and 12:00 AM and 24 cats (20.1%) between 12:00 AM and 6:00 AM.

### 4.9 Landing Surface

**FIGURE 11:**
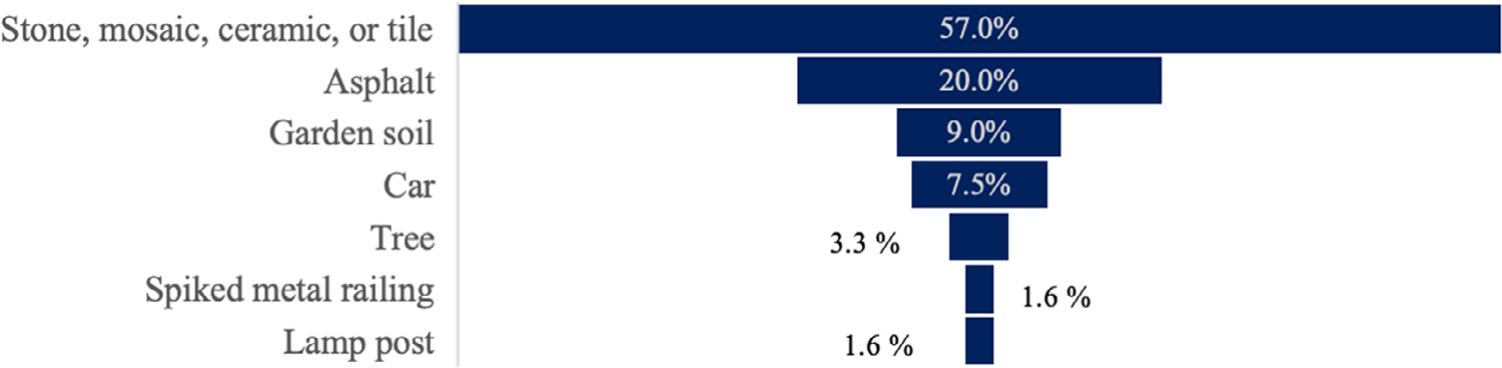
Landing surface frequency distribution of cats with HRS.

Regarding landing surfaces, 68 cats (57%) fell on stone, mosaic, ceramic or tile; 24 cats (20%) on asphalt; 11 cats (9%) on garden soil; 9 cats (7.5%) on cars; 4 cats (3.3%) on trees; 2 cats (1.6%) on spiked metal railings; and 2 cats (1.6%) on lamp posts (Figure 11). The Mann–Whitney *U* test comparing injury severity by surface showed a statistically significant difference (*p* = 0.003) (Figure S5), indicating that cats landing on hard surfaces sustained significantly more severe injuries (mean score 6.2) than those landing on soft surfaces (mean score 3.18).

### 4.10 Behavioural Findings

Only nine owners (7.5%) witnessed their cats falling. Approximately 3.3% of cats (four cases) had a history of HRS without injury. Interestingly, 93 cats (77.5%) had displayed risky behaviour, such as jumping on curtains. Additionally, 34 cases (28.3%) lived with another cat at home.

### 4.11 Soft Tissue Injuries

Injuries were categorized into soft tissue and hard tissue (orthopaedic) injuries. Soft tissue injuries included pneumothorax (20.7%), alveolar‐interstitial pattern (pulmonary haemorrhage/pulmonary contusion, 45%), reactive mesenteric lymphadenomegaly (17.5%), pleural effusion (3.3%), subcutaneous emphysema (3.3%), peritonitis (1.6%), pancreatitis (7.5%), free fluid in the abdominal cavity (22.5%), epistaxis (1.6%), rupture of the collateral ligament of the knee (0.8%), haematuria (7.5%), steatitis (3.3%), pulmonary atelectasis (10.8%), splenic haematoma (4.1%), pneumomediastinum (2.5%), partial muscle tear (0.8%), pneumoabdomen (1.6%) and abdominal hernia (0.8%). Microspleen was observed in 1.6% and pulmonary bulla in 0.8% of cats (Table 2).

**TABLE 2:** Soft tissue injuries of HRS patients.

Soft tissue injury	*n* (%)
Pulmonary haemorrhage/contusion	54 (45.0)
Free fluid in abdominal cavity	27 (22.5)
Pneumothorax	25 (20.7)
Reactive mesenteric lymphadenomegaly	21 (17.5)
Pulmonary atelectasis	13 (10.8)
Pancreatitis	9 (7.5)
Haematuria	9 (7.5)
Splenic haematoma	5 (4.1)
Pleural effusion	4 (3.3)
Subcutaneous emphysema	4 (3.3)
Steatitis	4 (3.3)
Pneumomediastinum	3 (2.5)
Peritonitis	2 (1.6)
Epistaxis	2 (1.6)
Pneumoabdomen	2 (1.6)
Microspleen	2 (1.6)
Abdominal hernia	1 (0.8)
Partial muscle tear	1 (0.8)
Collateral ligament rupture (knee)	1 (0.8)
Pulmonary bulla	1 (0.8)

### 4.12 Hard Tissue (Orthopaedic) Injuries

As shown in Tables 3, 4, 5, 6, hard tissue (orthopaedic) injuries included head fractures (three mandible fractures, two tooth fractures, two skull fractures, 5.8%), vertebral column fractures (one T10, two T12, two T13, two L1, one S2; 6.6%), anterior limb fractures (one scapula, 10 humerus, seven radius, eight ulna, eight carpal/metacarpal/digit; 28.3%) and posterior limb fractures (11 hip, 21 femur, 14 tibia, seven fibula, seven tarsus/metatarsus/digit, one sacrum; 50.8%). Additional injuries included rib fractures (5%), sacroiliac luxation (2.5%), hip joint luxation (1.6%), carpal joint luxation (1.6%), temporomandibular joint luxation (5%), humeroulnar luxation (2.4%), mandibular symphysis luxation (0.8%), intervertebral luxation (5.8%), tarsal joint luxation (0.8%) and shoulder joint luxation (0.8%). Hip fractures involved the ischium (36.3%), ilium (54.5%) and pubis (9%). Intervertebral displacement occurred between T10‐T11 (14%), T11‐T12 (14%), T12‐T13 (14%), T13‐L1 (42.8%) and L1‐L2 (14%). There were two total skull fractures, one involving the parietal bone and one the temporal bone. The cat with the parietal bone fracture was euthanized due to injury severity.

**TABLE 3:** Hip injuries of HRS patients.

Hip fracture location	*n* (%)
Ilium	6 (54.5)
Ischium	4 (36.3)
Pubis	1 (9.0)

**TABLE 4:** Intervertebral displacement site of HRS patients.

Intervertebral displacement site	*n* (%)
T10–T11	1 (14.0)
T11–T12	1 (14.0)
T12–T13	1 (14.0)
T13–L1	3 (42.8)
L1–L2	1 (14.0)

**TABLE 5:** Fractures site of HRS patients.

Region of fracture	Open fracture (*n* [%])	Closed fracture (*n* [%])	Complete fracture (*n* [%])	Incomplete fracture (*n* [%])
**Head**	0	7 (100)	6 (85.7)	1 (14.3)
**Vertebral column**	0	8 (100)	5 (62.5)	3 (37.5)
**Scapula**	0	1 (100)	1 (100)	0
**Humerus**	0	10 (100)	7 (70)	3 (30)
**Radius**	0	7 (100)	5 (71.5)	2 (28.5)
**Ulna**	0	8 (100)	3 (37.5)	5 (62.5)
**Carp/metacarp/digits**	1 (12.5)	7 (87.5)	6 (75)	2 (25)
**Hip**	0	11 (100)	8 (72.8)	3 (27.2)
**Femur**	2 (9.5)	19 (90.5)	18 (85.7)	3 (14.3)
**Tibia**	0	14 (100)	8 (57.1)	6 (42.9)
**Fibula**	0	7 (100)	4 (57.1)	3 (42.9)
**Tars/metatars/digits**	1 (14.3)	6 (85.7)	5 (71.5)	2 (28.5)
**Ribs**	0	6 (100)	6 (100)	0

**TABLE 6:** Clinical outcomes, survival rates, and injury scores.

Result	Died	Euthanized	Survived (including euthanized)	Survived (excluding euthanized)	No specific injury
**Patients (*n*)**	9	4	107	111	7
**Total %**	7.5	3.5	89.2	92.5	5.8

**FIGURE 12:**
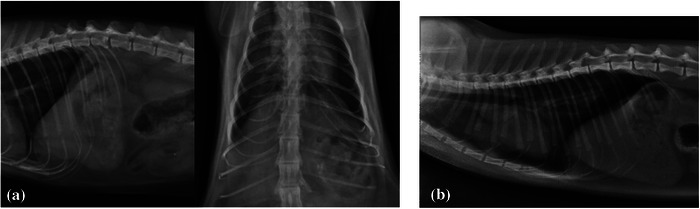
(a) There is a fracture in the left transverse process, caudal end plate and part of the body of T13 vertebra with dorsal displacement of fracture fragment associated with narrowing of spinal canal at fracture site. (b) Cranial cardiac silhouette is obscured by soft tissue opacity and caudal part is elevated from sternum, which illustrate moderate to severe alveolar lung pattern due to contusion and haemorrhage and moderate pneumothorax; also a fracture is noted in the 13th left rib.

**FIGURE 13:**
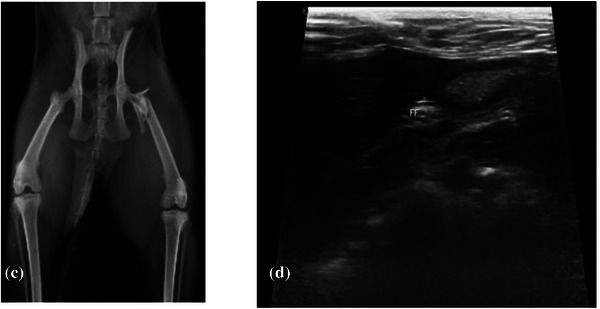
(c) There is a comminuted fracture in proximal third diaphysis of left femur with multiple fracture fragments; also, increased soft tissue volume around left femur is noted. (d) Slight amount of abdominal free fluid around liver in the near field is presented.

Two representative cases of HRS injuries are shown in Figures 12 and 13 (radiographs and sonographs, respectively).

## 5 Discussion

The mean age of cats was 1.3 years, consistent with earlier reports (Çatalkaya et al. 2022; Karabağlı et al. 2024; Oxley and Montrose 2016), though higher values (3–4 years) have been noted in smaller cohorts (Robinson 1976; Xiang 2018). Mortality was 7.5% (mean score 15) and euthanasia 3.3% (mean score 10.75). Overall survival reached 89.2% or 92.5% when excluding euthanized cases, comparable to prior studies (Duhautois et al. 2010; Karabağlı et al. 2024; Lynch 2017; Oxley and Montrose 2016; Vidal et al. 2024; Warner and Demling 1986). Most falls occurred from the third floor (29.1%), with a mean of 3.33 floors, within the common third–fourth floor range (Duhautois et al. 2010; Oxley and Montrose 2016; Vidal et al. 2024; Warner and Demling 1986; Xiang 2018).

Males comprised 57.5% and females 42.5% of cases, with no significant sex effect. Intact males were most frequent (38.3%), followed by intact females (27.5%), neutered males (19.1%) and spayed females (15%). Similar patterns have been reported (Karabağlı et al. 2024; Warner and Demling 1986; Xiang 2018). The predominance of neutered males over intact males may reflect cultural practices favouring male neutering due to lower costs. Domestic Shorthair was the most common breed (74.1%).

Falls peaked in warmer months and were least frequent in December (3.42%), consistent with other reports (Duhautois et al. 2010; Karabağlı et al. 2024; Tüzün and Sağlam 2009; Vidal et al. 2024; Xiang 2018). Hard surfaces accounted for 81.6% of landings, associated with more severe injuries (*p* = 0.003), as also noted in other researches (Karabağlı et al. 2024). Risky behaviours were reported in 77.5% of cats, while food availability was not a factor (∼90% of patients had full food bowls).

No diaphragmatic hernia (DH) was observed, consistent with Duhautois et al. (2010) and Robinson (1976), likely due to simultaneous thoracic and abdominal pressure changes, unlike vehicular trauma where DH occurs in ∼85% (Kazemi et al. 2022). As HRS is the second most common trauma after road accidents, diaphragmatic ruptures with unclear history may indicate traffic injury.

Pulmonary haemorrhage/contusion was the most frequent soft tissue lesion (45%), aligning with Warner and Demling (1986) (61.2%) and Robinson (1976) (36.1%), but higher than Oxley and Montrose (2016) (7%) and Pratschke and Kirby (2002). Femoral fracture was the most common orthopaedic injury (Duhautois et al. 2010; Oxley and Montrose 2016; Warner and Demling 1986), though tibial fractures dominated in some studies (Righi 2019; Vidal et al. 2024). Ilium was the most frequent pelvic fracture site (Çatalkaya et al. 2022). Spinal injuries mainly involved the thoracolumbar junction (Robinson 1976). The ratio of forelimb to hindlimb fractures was 1:1.55, similar to prior studies (Warner and Demling (1986)—1:1.63; Vidal et al. (2024)—1:1.6; Xiang (2018)—1:1.7; Çatalkaya et al. (2022)—1:1.6).

## 6 Conclusion

HRS is most common in young cats under 1 year of age. Although the overall survival rate was high (89.2%), outcomes depend on factors such as the surface of impact, type of injury and quality of post‐accident care. In this study, most falls occurred from the third floor. Preventive measures, including restricting behaviours predisposing to falls and securing windows and balconies with nets, are essential to reduce incidence. Soft tissue injuries, particularly pulmonary haemorrhage and contusion, were observed in nearly half of the cases. The most frequent dislocation was intervertebral (T13‐L1), and the most common orthopaedic injury was femoral fracture. Less frequent but severe complications, including intervertebral displacement, calvarial and vertebral fractures, warrant close monitoring with advanced imaging when indicated, as they can be life threatening. Higher injury severity scores were associated with increased risk of fatal outcomes. Hormonal factors may contribute, and further studies should investigate the role of sex hormones in affected cats.

## Author Contributions

**Parisa Haghi**: conceptualization, methodology, software, data curation, investigation, validation, formal analysis, visualization, writing – original draft. **Sarang Soroori**: conceptualization, methodology, software, data curation, investigation, validation, formal analysis, supervision, funding acquisition, visualization, project administration, resources, writing – original draft, writing – review and editing. **Mohammad Molazem**: conceptualization, data curation, investigation, validation, supervision, resources, writing – review and editing. **Mir Sepehr Pedram**: conceptualization, data curation, investigation, validation, supervision, resources, writing – review and editing. **Athena Salimi**: data curation, writing – review and editing. **Arezoo Ramezani**: data curation, writing – review and editing.

## Funding

The authors have nothing to report.

## Ethics Statement

This study is based on a review of clinical records already collected from the Veterinary Hospital of Tehran University. Since no new animal interventions or the use of live animals were involved, ethical committee approval wasn't necessary.

## Conflicts of Interest

The authors declare no conflicts of interest.

## Supporting information




**Figure S1**. Injuries in cats related to increasing fall height (P value = 0.061).
**Figure S2**. Injuries in cats related to increasing weights (p value = 0.089).
**Figure S3**. Association between sex and injury in cats (p value = 0.65).
**Figure S4**. Association between Reproductive status and injury in cats (p value = 0.295)

## Data Availability

The data that support the findings of this study are available on request from the corresponding author. The data are not publicly available due to privacy or ethical restrictions.
