# Supplementary material for: Radiographic and Ultrasonographic Study of the Etiology of High‐Rise Syndrome in Cats: A Retrospective Analysis
Source: Vet Med Sci. 2026 May 19;12(3):e71002. doi: 10.1002/vms3.71002 (PMC13185220; doi:10.1002/vms3.71002)

**Appendix**


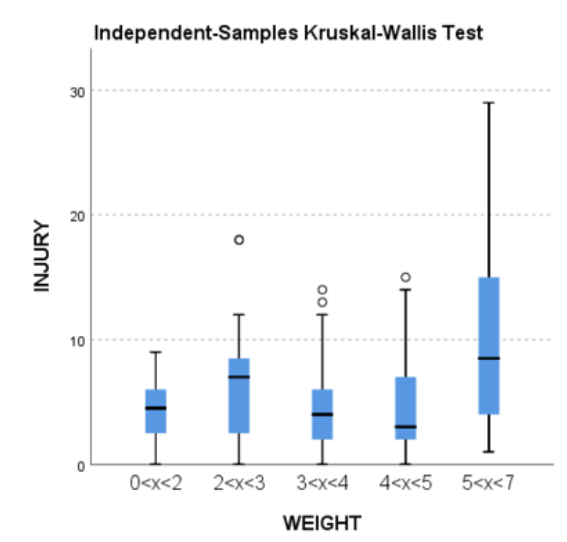

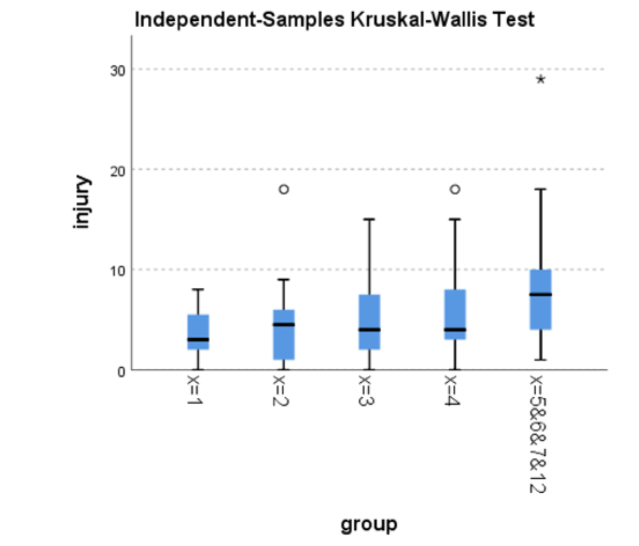


Figure S1. Injuries in cats related to

increasing fall height (P value = 0.061)

Figure S2. Injuries in cats related to

increasing weights (p value= 0.089)


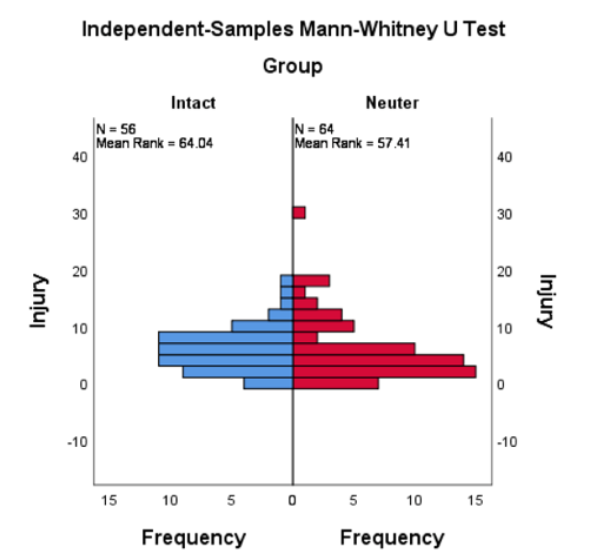

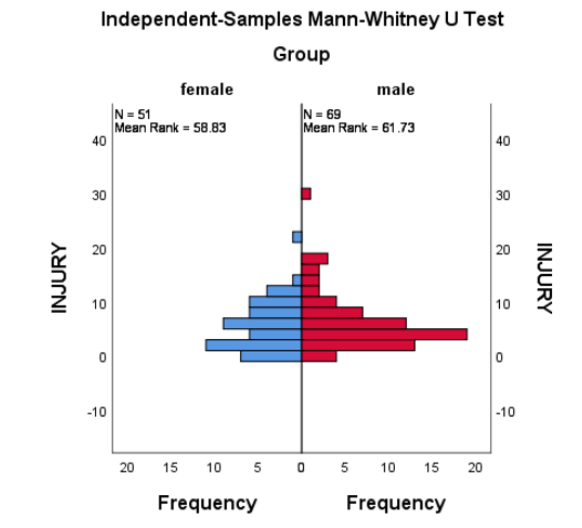


Figure S5. Association between

different surfaces and injury in cats (P value= 0.003)

Figure S4. Association between

Reproductive status and injury in cats (p value= 0.295)

Figure S3. Association between

sex and injury in cats (p value= 0.65)


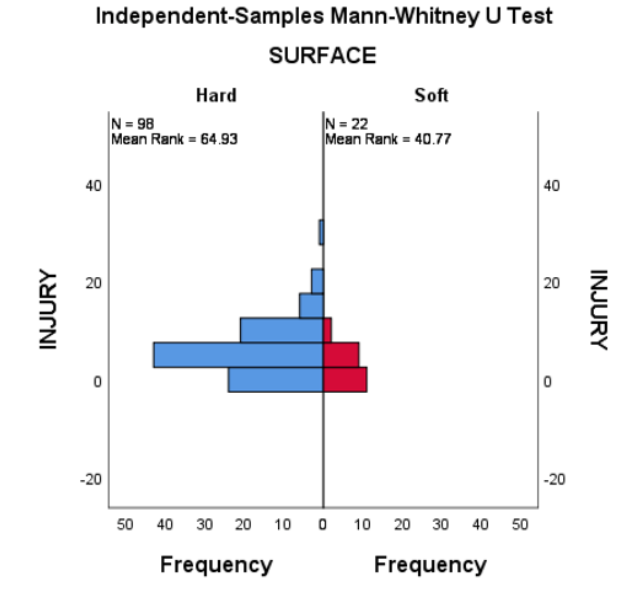

Supplement: Supplementary file 1 — Figure S1. Injuries in cats related to increasing fall height (P value = 0.061). Figure S2. Injuries in cats related to increasing weights (p value = 0.089). Figure S3. Association between sex and injury in cats (p value = 0.65). Figure S4. Association between Reproductive status and injury in cats (p value = 0.295) [file VMS3-12-e71002-s001.docx]
